# Fatal cerebral myiasis secondary to squamous cell carcinoma: case report and scoping review

**DOI:** 10.1590/S1678-9946202668010

**Published:** 2026-01-30

**Authors:** Paulo Henrique Alves Togni, Ernani Alves de Oliveira, Roscicler Pereira de Sousa, André Luís Santos Vaz Leite, Lucas Eiji Adachi Oliveira, Guilherme Augusto Paro, Julia de Campos Preto

**Affiliations:** 1 Centro Universitário Padre Albino, Departamento de Radiologia e Diagnóstico por Imagem, Catanduva, São Paulo, Brazil; 2 Centro Universitário Padre Albino, Departamento de Neurocirurgia, Catanduva, São Paulo, Brazil; 3 Centro Universitário Padre Albino, Catanduva, São Paulo, Brazil; 4 Faculdade de Medicina de Catanduva, Catanduva, São Paulo, Brazil

**Keywords:** Myiasis, Infection, Brain, Osteomyelitis, Abscess

## Abstract

Cerebral myiasis is an exceptionally rare condition caused by infestation with dipteran larvae, with only 20 cases reported in the literature to date. A 78-year-old man presented with anorexia, vomiting, and fever. Physical examination revealed a 7 × 8 cm ulcerated scalp lesion with a necrotic base, purulent discharge, a foul odor, and numerous larvae. Computed tomography demonstrated an osteolytic frontal bone defect accompanied by pneumocephalus and subcutaneous emphysema. The larvae were manually removed, an iodoform dressing was applied, and intravenous ceftriaxone therapy was initiated. Progressive neurological decline prompted repeat imaging, which revealed frontal and parietal cerebritis with abscess formation. Surgical debridement was performed to remove necrotic tissue. Histopathological analysis showed moderately differentiated squamous cell carcinoma with acute osteomyelitis, and cultures yielded multidrug-resistant *Pseudomonas aeruginosa*. Despite targeted antibiotic therapy and intensive supportive care, the patient died. This case highlights the significant morbidity and mortality associated with cerebral myiasis, particularly when complicated by underlying malignancy and multidrug-resistant infection. Early recognition, prompt surgical intervention, and pathogen-directed antimicrobial therapy are crucial, while comprehensive multidisciplinary management remains essential to optimize outcomes in this life-threatening condition.

## INTRODUCTION

Myiasis is defined as the infestation of living vertebrates by larvae of the order Diptera and can be classified according to parasitological or clinical criteria^1,2^. Alternatively, it may also be categorized based on ecological or anatomical characteristics^1,2^. Patton^3,4^ divided myiasis-causing flies into three parasitological groups: obligate, facultative, and accidental. Obligate parasites require living tissue for larval development, whereas facultative parasites typically develop in decaying organic matter but may occasionally invade living tissue. In accidental parasitism, eggs or larvae are inadvertently ingested or inhaled with contaminated food^1^. Clinical classification is based on the anatomical site of infestation, first proposed by Bishopp^5^ and later modified by James^6^ and Zumpt^7^, encompassing categories such as blood-sucking, tissue-destroying, subdermal, gastrointestinal, and urogenital myiasis, as well as infestations of the head passages (e.g., ear, nose, and throat)^1,2,4^.

Cerebral myiasis is exceptionally rare, with the first case reported in 1939 by Froomin and Kaznelson^8^. Since then, only 20 cases have been previously documented in the literature. Despite its rarity, cerebral myiasis poses a severe clinical challenge, often resulting in significant neurological impairment, high morbidity, and, in some cases, mortality^9^.

Although individual case reports provide valuable insights into the clinical presentation and management of cerebral myiasis, no comprehensive synthesis of the available evidence exists to support clinical decision-making. Important questions remain regarding its epidemiology, risk factors, and optimal therapeutic approaches.

Thus, a scoping review was conducted to systematically map the available research on intracerebral myiasis, identify trends in reported cases, and highlight existing knowledge gaps. This review was guided by the following research question: What does the literature reveal about the clinical presentation, management, and outcomes of patients diagnosed with intracerebral myiasis?

Despite its rarity, the published reports of intracerebral myiasis span a broad temporal range—from 1939 to the present—and encompass diverse etiologic contexts and clinical presentations, including post-traumatic, postoperative, cutaneous head-and-neck malignancy, and pediatric cases. This review covers both historical and contemporary records, which collectively consolidate the clinical spectrum observed to date^8^–^27^.

### Ethics

This study was approved by the Research Ethics Committee (CEP) of the Padre Albino University Center, under CAAE Nº 68395723.7.0000.5430, and Substantiated Opinion Nº 6.000.608.

## MATERIALS AND METHODS

The protocol for this scoping review was developed a priori following the Preferred Reporting Items for Systematic Reviews and Meta-Analyses for Scoping Reviews (PRISMA-ScR) guidelines^28^. The initial draft was reviewed by the research team and revised accordingly before registration. The final protocol was prospectively registered with the Open Science Framework (OSF) in March 2025^29^. Any deviations from the protocol during the review process are documented and justified in the Results section, ensuring transparency and reproducibility.

### Literature search

A comprehensive literature search was conducted across PubMed, SciELO, and BVS on January 26, 2025, with no language or publication date restrictions, to maximize inclusivity of all relevant studies on cerebral myiasis (CM). The search strategy was developed by an experienced librarian and further refined with team discussion, using a combination of controlled vocabulary terms (MeSH) and non-controlled keywords in multiple languages.

The PubMed search strategy included the following combination of MeSH terms and keywords: ("Cerebral Myiasis" OR "intracerebral" OR "Brain Myiasis" OR "Intracranial Myiasis" OR "NeuroMyiasis" OR "Central Nervous System Myiasis") AND ("Myiasis"[MeSH] OR "Parasitic Infestation"[MeSH] OR "Fly Larvae Infestation" OR "Maggot Infestation" OR "Diptera Infestation" OR "Larval Infection" OR "Infection by Fly Larvae" OR "Parasitic Larvae" OR "Myiasis Infection" OR "Grubs Infestation" OR "Grubs" OR "Myiasis").

Additionally, manual reference checks of the included studies were performed to identify potentially overlooked articles and relevant gray literature.

### Selection process

Eligibility criteria were established using the PCC framework: population (P): patients diagnosed with intracerebral myiasis; concept (C): clinical presentation, management, and outcomes; context (C): published case reports, case series, and observational studies.

All records were imported into Rayyan (Rayyan Systems Inc., Cambridge, MA, USA) for screening^30^. Two independent reviewers assessed titles and abstracts, with discrepancies resolved by means of discussion and consensus.

Exclusion criteria comprised non-cerebral myiasis (e.g., ophthalmic, optic, nasopharyngeal), non-dipteran infestations (e.g., gnathostomiasis, nematode larval migration; two papers excluded for this reason), duplicates, and records lacking sufficient case-level information.

Data extraction was performed using a pre-piloted form capturing study identifiers, patient demographics, clinical presentation, underlying conditions, characteristics of the infestation, diagnostic methods, therapeutic interventions, outcomes, and study design features. The extracted variables were informed by, and subsequently compared with, those reported by Cuestas *et al.* ^31^, enabling identification of methodological similarities and differences and aligning this review with established reporting standards for myiasis in malignant wounds.

Despite the use of a standardized data extraction form, several studies lacked complete information for key variables (e.g., larval species identification, patients’ functional baseline), which constrained the comprehensiveness of the synthesized findings.

## RESULTS

The initial search on the SciELO platform yielded a low number of results, which, upon further analysis, suggested an artificial restriction in article retrieval. To address this limitation, search terms were refined by incorporating additional focused descriptors. In PubMed, the addition of the ‘[tiab:∼0]’ operator after certain keywords reduced the number of retrieved records from 65 to 14, underscoring the importance of maintaining a broader initial search to ensure comprehensive coverage.

The final SciELO strategy was therefore simplified to a core set of terms: ‘Myiasis’ OR ‘Cerebral Myiasis’ OR ‘Brain Myiasis.’

**Figure 1:**
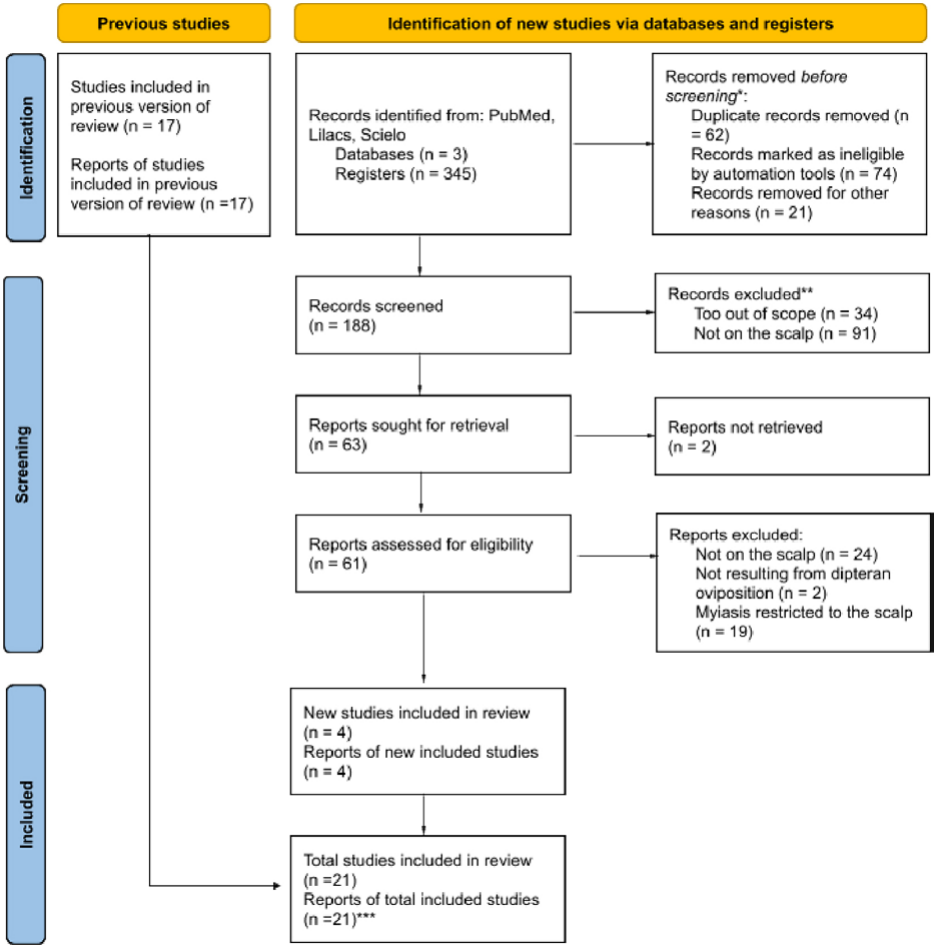
PRISMA flow diagram illustrating the selection process of the included studies; *automation tools labeled "keywords" were used in Rayyan to identify articles containing the words "myiasis," "neuromeningeal," or "larvae" that did not refer to humans; despite the use of automation, human analysis was always performed before any exclusion; **the same automation tool was used to help identify articles in which the myiasis did not occur in the brain; other reports that substantially deviated from the central topic were also excluded (essentially those addressing therapies, diagnostic keys, or epidemiological analysis rather than myiasis cases) and categorized as "not related/out of scope"; ***present case included.

To further supplement the electronic searches, an exploratory search was conducted on the Research Rabbit platform^32^. In total, 20 studies were deemed pertinent and included for full-text review and data extraction (Figure 1).

## CASE REPORT

A 78-year-old Caucasian male was admitted to the emergency department, accompanied by a family member, with complaints of anorexia, vomiting, and fever that had begun earlier that day. He reported living alone and being independent in both basic and instrumental activities of daily living. His only known medical condition was systemic arterial hypertension—confirmed by his daughter—and was taking 50mg of Losartan once every morning.

On physical examination, the patient appeared confused and uncommunicative, with a mildly pale complexion and clinical signs of dehydration, but was afebrile. Inspection of the scalp revealed an ulcerated lesion in the left frontal region extending to the parietal border, measuring approximately 7.0 cm × 8.0 cm. The lesion exhibited irregular, raised, hyperemic margins and a necrotic base with purulent discharge and a strong fetid odor. Numerous fly larvae (myiasis) were observed within the wound, actively consuming necrotic tissue—an observation consistent with the patient's report that the lesion had been progressively enlarging over the past year.

Initially, larvae were manually removed using anatomical forceps and irrigation with 0.9% saline solution. Upon identifying bone exposure, an occlusive iodoform dressing was applied. Based on the initial presentation, the primary hypothesis was cutaneous myiasis. However, after larval removal and identification of bone exposure and involvement, the possibility of extension into the brain parenchyma was considered. No alternative differential diagnoses were proposed. Laboratory tests and a computed tomography (CT) scan of the skull were requested for further evaluation.

The complete blood count (CBC) revealed a hemoglobin level of 11.3 g/dL (reference range for older adult males: 13.0–17.0 g/dL), a hematocrit of 32.7% (reference: 38.8%–50.0%), and leukocytosis with a total leukocyte count of 19,400/µL (reference: 4,000–11,000/µL), with marked neutrophilic predominance (89.8%, reference: 40%–70%; absolute neutrophils count: 17,400/ µL). Serum sodium was 133 mmol/L (reference: 135–145 mmol/L), indicating mild hyponatremia, and blood urea nitrogen was elevated at 72 mg/dL (reference: 7–20 mg/dL).

**Figure 2:**
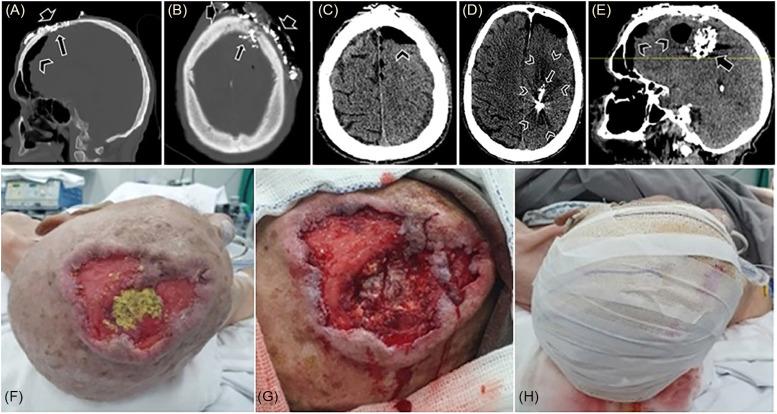
First CT scan: bone window, sagittal (A) and axial (B) planes – osteolytic lesion in the frontal bone (long arrow) establishing communication between the meningeal membranes and the external environment through an extensive ulcerated lesion (short arrow) in the skin and subcutaneous tissue. Pneumocephalus (arrowhead); (C) parenchymal window, pneumocephalus (arrowhead). Second CT scan: parenchymal window: (D) axial plane – hypodense lesions in the left frontal and parietal lobes, suggesting organized cerebritis/abscess with possible areas of necrosis (arrowhead); multiple metallic-density particles intermingling these tissues, representing iodoform (long arrow); (E) sagittal plane – iodoform particles (arrow) and pneumocephalus (arrowhead); (F) extensive irregular wound measuring 7.0 cm × 8.0 cm, with raised and erythematous edges; presence of gauze with Iodoform in the region of greatest excavation, corresponding to the osteolytic lesion; (G) post-debridement local appearance showing exposed meningeal membranes; (H) local occlusive dressing with Rayon, Rifocin, and Dersani gauze.

The CT scan revealed an osteolytic defect in the left frontal bone, establishing communication between the meningeal membranes and the external environment through an ulcerated lesion involving the skin and underlying tissues in the frontal region, predominantly left of the midline. Additional findings included pneumocephalus, subcutaneous emphysema, and multiple metallic-density particles interspersing the nervous tissue, consistent with iodoform (Figure 2). The patient was subsequently admitted to the neurosurgery ward for continued care, and intravenous antibiotic therapy with ceftriaxone (1 g every 12 h) was initiated.

The patient initially received general care, including respiratory physical therapy and routine wound care with regular dressing changes. On the fifth day of hospitalization, the patient developed drowsiness, asthenia, anorexia, constipation, and urinary retention. Laboratory tests were ordered, along with a chest radiograph, nasoenteral tube placement, and delayed bladder catheterization. Results showed a total leukocyte count of 14,600/µL with 82.1% neutrophils (absolute neutrophils count: 11,986/µL), urea of 94 mg/dL, and a chest radiograph without significant abnormalities.

On the ninth day of hospitalization, despite ongoing supportive measures, the patient exhibited a continued decline in mental status, prompting a repeat cranial CT for preoperative evaluation (Figure 2). Compared to the previous scan, new hypodense lesions were observed in the left frontal and parietal lobes, accompanied by erasure of the ipsilateral cortical sulci, suggesting cerebritis with an organizing abscess and possible necrotic areas. Consequently, the patient underwent surgery, during which the lesion was debrided and irrigated with Hexamidine and 0.9% saline solution. The necrotic tissue, heavily colonized by Iodoform and putrefying myiasis, was thoroughly removed, resulting in complete abscess evacuation. Hemostasis was achieved, followed by the application of an occlusive dressing using Rayon, Rifocin, and Dersani gauze (Figure 2). Samples were submitted for histopathological analysis, bacterioscopy, and antibiogram.

**Figure 3:**
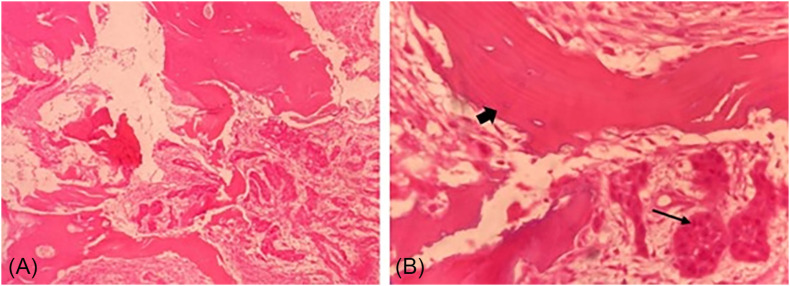
Anatomopathology: (A) bone fragment showing invasion by a moderately differentiated squamous cell carcinoma; neoplastic cells exhibit pleomorphism with intercellular bridges and infiltrate the bone tissue; (B) bone tissue (thick arrow) and nests of moderately differentiated malignant squamous cells (thin arrow).

Pathology showed a moderately differentiated and invasive squamous cell carcinoma in the bone tissue, along with acute exudative osteomyelitis (Figure 3). The microbiological culture identified *Pseudomonas aeruginosa*, with its antimicrobial susceptibility profile determined by automated microdilution.

The isolate was resistant to cefepime (MIC >8 µg/mL), ceftazidime (MIC >8 µg/mL), piperacillin-tazobactam (MIC >16 µg/mL), ciprofloxacin (MIC >1 µg/mL), and levofloxacin (MIC >1 µg/mL). Resistance to broad-spectrum β-lactams, combined with fluoroquinolone resistance, suggests the possible expression of mechanisms such as extended-spectrum β-lactamases (ESBL) or carbapenemases.

Carbapenems showed an intermediate profile with imipenem (MIC undetermined) and meropenem (MIC 8 µg/mL), indicating the need for dose optimization to enhance clinical efficacy. In contrast, the isolate remained susceptible to colistin (MIC ≤2 µg/mL), amikacin (MIC ≤8 µg/mL), and tobramycin (MIC ≤2 µg/mL), suggesting potential treatment options with aminoglycosides or polymyxins, depending on pharmacokinetic and pharmacodynamic parameters at the infection site.

On the first postoperative day (Table 1), the patient's level of consciousness remained depressed. Antibiotic therapy was switched from Ceftriaxone (1g IV every 12 h) to Vancomycin (500mg IV every 12 h) after 12 days of treatment. On the fifth postoperative day, the patient developed an episode of desaturation, which was stabilized with an oxygen mask. However, the patient's sensory decline persisted, prompting a palliative care consultation.

**Table 1:** Temporal progression of the patient's clinical and laboratory parameters.

Day	Clinical data	Laboratory results	Interventions
**Pre-OP Status (03/02)**	--	Hb: 11.1 g/dl	--
		Glucose: 125 mg/dl	
		Leuco: 14,600/µL (82.1% neutrophils)	
		K: 4.2 mmol/L	
		Na: 136 mmol/L	
		Platelets: 136.000/uL	
		Clotting time: 7 min	
		TTPa: 15.6 (73% ativ. and INR 1.22)	
		DUKE: 2 min	
		Urea: 94 mg/dL	
		Creat: 0.70 mg/dl	
**Day 1 Post-OP (03/09)**	Moderate pallor (2+/4+), hydrated. Bilateral vesicular breath sounds audible, with scattered wheezes. BP: 120/60, HR: 107. No other systems altered.	Na: 126 mmol/L	Antibiotic switch to Vancomycin (500 mg IV every 12 hours)
		K: 4.6 mmol/L	
		Glucose: 113 mg/dl	
		Leuco: 17,200/µL (79.3% neutrophils)	
		Creat: 0.60 mg/dl	
		Urea: 53 mg/dL	
**Day 2 Post-OP (03/13)**	Emaciated and cachectic. Presents an ulcerated lesion in the sacral region and diffuse hematomas in both upper limbs. No signs of inflammation. Indwelling urinary catheter with dark yellow urine output. Glasgow 11/15.	Hb: 8.4 g/dL	Enteral nutrition at 20 mL/h. Medication in intravenous infusion of Sodium Chloride at 2 mL/h.
		Leuco: 21,100/µL (86% neutrophils)	
		K: 3.6 mmol/L	
		Na: 128 mmol/L	
		Glucose: 160 mg/dl	
		Urea: 75 mg/dL	
		Creat: 0.80 mg/dl	
**Day 3 Post-OP (03/14)**	Pale (++/4+), hydrated, on high-flow mask 15L/min. Afebrile and without signs of infection. Glasgow 10/15. SPO2: 95%. HR 115.	Hb: 8.4 g/dL	High-concentration oxygen mask at 10L/min.
		Leuco: 17,200/µL (89.8% neutrophils)	
		K: 3.7 mmol/L	
		Na: 143 mmol/L	
		Urea: 52 mg/dL	
		Creat: 0.40 mg/dl	

* The table outlines the patient's clinical and laboratory findings throughout the initial postoperative period, highlighting management decisions and subsequent interventions based on the patient's evolving condition; Hb = Hemoglobin; Leuco = Leukocytes; Na = sodium; K = potassium; Creat = creatinine.

The patient was transferred to the palliative care team at a secondary hospital, where Meropenem was added to the antibiotic regimen. Sodium levels were corrected, and a high-flow oxygen mask (10 L/min) was maintained, resulting in a 96% oxygen saturation (SpO2). Although some transient improvements were observed, such as mild stabilization of diuresis and wound healing, the overall prognosis persisted. The patient remained non-communicative, exhibiting limited responsiveness to painful stimuli, and continued to require intensive supportive care, including nutritional supplementation and secretion management over the subsequent 10 days. Ultimately, the patient died.

## DISCUSSION

Intracerebral myiasis is an extremely rare entity, with only 20 cases reported worldwide to date, according to the most recent literature review conducted in 2024 by Ramón-Cuellar *et al.* ^10^. This is the fifth documented case in Brazil.

Human myiasis is globally distributed, with higher prevalence in developing countries—particularly in humid tropical and subtropical regions—where greater species diversity and abundance are observed^1,2,33^. The most significant risk factors for myiasis include poor hygiene and low socioeconomic status. Additionally, immunosuppression, diabetes, psychiatric disorders, advanced age, delayed access to specialized medical care, and alcoholism are also recognized as predisposing factors^1,2,11,31,33^ (Table 2).

**Table 2:** Reported cases of cerebral myiasis.

Article	Country/Origin	Year	Sex	Age	Etiology	Location	Larval Type	Treatment	Complication	Death
Froomin *et al*.^8^	Russia	1939	Female	50	No data	No data	No data	No data	No data	No data
Semenov *et al.* ^12^	Russia	1969	Male	4	Unknown	Occipital	*Hypoderma lineatum*	No data	Death	Yes
Zucoloto and Ross^13^	Brazil	1971	Male	40–60	Unknown	Frontal	Unidentified	No data	No data	Yes
Rossi and Zocoluto^14^	Brazil	1973	Female	0.42	Unknown	Frontoparietal	*Dermatobia hominis*	No treatment	Death	Yes
Gilly *et al*.^15^	France	1976	Male	7	Post-operative of hematoma drainage	Frontotemporoparietal	*Hypoderma bovis*	RS, D, and AT	Death	Yes
Pouillaude *et al*.^16^	France	1980	Male	6	Unknown	Temporal	*H.bovis*	RS, D, and AT	Death	Yes
François *et al*.^17^	France	1987	Male	9	Secondary to blepharitis	Cerebellar	*Hypoderma spp.*	AT	Hydrocephalus	No
Kalelioglu *et al*.^18^	Türkiye	1989	Male	8	Unknown	Parieto–occipital	*H.bovis*	RS, D, and AT	No	No
Cheshier *et al*.^19^	United States	2007	Male	75	Angiosarcoma of the scalp	Frontal	*Phaenicia sericata*	RS, D, and AT	Death after months	Yes
Marco de Lucas *et al*.^20^	Spain	2008	Male	11	Post traumatic	Frontal–temporal–occipital	*D. hominis*	Sur. at three years, 11 years, and no other	Refractory seizures	No
Terterov *et al*.^21^	United States	2010	Male	42	Posttraumatic and immunosuppression (HIV)	Frontal	Unidentified	RS, D, and AT	No	No
Holanda *et al.* ^22^	Brazil	2015	Male	85	Associated to past right ocular enucleation	Frontal	Unidentified	RS, D, and AT	Sepsis	Yes
Giri *et al*.^23^	India	2016	Male	38	Associated with Artificial Cranioplasty Flap	Frontotemporoparietal	Nebulus Domestic fly	RS, D, and AT	No	No
Navarro and Alves^24^	Brazil	2016	Male	36	Postoperative complication	Frontotemporal	Unidentified	RS, D, and AT	Cognitive sequelae	No
Aggarwal and Maskara^25^	India	2017	Male	26	Posttraumatic	Frontoparietal	Unidentified	RS, D, and AT	No	No
Mariottiz Acuña *et al.* ^9^	Colombia	2017	Male	45	Unknown	Frontoparietal	Unidentified	RS, D, and AT	Death after months	Yes
Curzi *et al.* ^11^	Italy	2019	Male	72	Ulcerated basal cell carcinoma	Frontal	*Sarcophaga carnaria*	RS, D, and AT	Death after two months	Yes
Deo *et al*.^26^	India	2023	Male	64	Secondary to Burr Hole Evacuation	Temporal	*Sarcophaga carnaria*	D and AT	No data	No
Algahtany *et al*.^27^	Africa	2022	Male	24	Posttraumatic	Center temporal and cerebellum	Undenitified	Superficial debridement and AT	No data	No
Ramón-Cuellar *et al*.^10^	Colombia	2024	Female	74	Secondary to wound dehiscence post MVD.	Cerebellar	*Cochliomya hominivorax*	RS, D, and AT	No	No
This study	Brazil	2024	Male	78	Squamous cell Carcinoma	Frontoparietal	Unidentified	RS, D, and AT	Death	Yes

RS = removal surgery; D = debridement; AT = antibiotic treatment; sur = surgery; MVD = microvascular decompression; cases with age listed as 40–60 years indicate unconfirmed exact age, while 0.42 years corresponds to approximately five months; Unknown = cases in which the source of infestation was not determined; No data = missing information in the original report. The table highlights the diversity of etiologies, therapeutic approaches, and prognostic outcomes associated with intracerebral myiasis within all cases reported yet.

Based on our review, the age of patients affected by cerebral myiasis varied from five months to 85 years, with a mean of 37.7 years. Regarding gender, 18 out of 21 cases (85.7%) occurred among men. Reported symptoms include fever, chills, varying degrees of consciousness impairment, intracranial hematoma, motor deficits, convulsions, extrapyramidal effects, secondary infections, and intracranial hypertension with hydrocephalus^1,2,11,15,17,25,27^. Local symptoms may include pruritus, pain, bloody discharge, and the characteristic sensation of crawling or movement beneath the skin^1,11,31^. Symptom onset can be delayed for days to years, particularly among patients with dementia^11^. Additionally, advanced age alone may contribute to delayed clinical manifestation, as seen in this case, in which local symptoms were neglected.

Cutaneous involvement is the most common presentation of myiasis. In this case, the condition originated from a neglected scalp lesion that, upon histopathological examination, was diagnosed as a squamous cell carcinoma with bone tissue infiltration^19,25,27,31,34^. Notably, only Curzi *et al.* ^11^ in 2021 have reported squamous cell carcinoma as a risk factor for cerebral myiasis^31^. Sesterhenn *et al*.^35^ reported 20 cases of cutaneous myiasis associated with malignant head-and-neck lesions, seven (35.0%) of which involved preexisting squamous cell carcinoma. No single larval species predominated in those case, although *Lucilia sericata* was the most frequently identified.

According to the literature, the frontal lobe is the most frequently affected site, followed by the temporal lobe^11,16,19,20,23,24^. Deep parenchymal involvement and advanced age have been consistently associated with a poor prognosis^11^. The mortality so far reached 47.6%, representing a 61.9% relative increase compared with the mortality rate previously reported by Ramón-Cuellar (29.4%)^10^.

Despite prolonged exposure of nervous tissue, secondary infections, whether encephalitis (with or without abscess formation) or meningitis, were rare, with only four cases reported^19,20,27,35^. Although larvae are known to harbor a broad spectrum of bacteria capable of intensifying the local inflammatory response^35^, it is assumed that maggot infestation may exert a protective effect on exposed tissues. This occurs by means of mechanical coverage of the wound surface and digestion of necrotic tissue^1,21,23,26,35^. During the First World War, larvae of the Calliphoridae family, including *Lucilia sericata* and *Phormia regina*, were used for debriding wounds and osteomyelitis due to their saprophagous habits^35^. Pneumocephalus was another exceptional finding, described only in three cases^23,27,35^. No reports of osteomyelitis were found in the literature.

Various approaches have been described for larval removal^35^. Except for Algahtany *et al.* ^27^, who opted for conservative management, all other cases underwent surgical intervention involving larval removal, debridement of necrotic tissue, wound irrigation, and, when feasible, reconstruction of the affected area. These procedures were combined with broad-spectrum intravenous antibiotic therapy, with or without antifungal, antiparasitic, or anticonvulsant medications^9,11,19,21,23,26,27^. Furthermore, one of the most critical components of treatment is the consistent daily maintenance of dressings and wounds, along with maintaining overall patient hygiene, as these measures are key determinants in reducing infection risk.

Unfortunately, the exact larval species could not be identified in this case. However, considering the endemic species in the patient's geographical region and the known predisposing factors—such as advanced age, an exposed wound, and poor hygiene—it is most likely that *Cochliomyia hominivorax* was involved, as it is the primary causative agent of myiasis in Brazil^1^. Identifying the species, and consequently its life cycle, is crucial for understanding the mode of infestation, estimating its duration, predicting its progression, and implementing effective preventive measures^35^.

### Limitations

This scoping review has some inherent limitations. First, the inclusion of only published case reports and series raises the possibility of publication bias, since unusual or severe presentations are more likely to be reported, potentially limiting generalizability. Second, the scarcity and heterogeneity of available data— particularly regarding larval species identification, patients’ functional baseline, and standardized outcomes—restricted the comprehensiveness of the synthesis. Finally, as with all scoping reviews, the methodology prioritizes breadth over depth, aiming to map the existing literature rather than to generate quantitative effect estimates.

Moreover, this study did not receive external funding, which eliminates potential funding-related bias but also limits the availability of resources that might have enhanced data collection and analysis. Similarly, the authors declare no conflicts of interest, ensuring impartiality in the review process.

Despite these limitations, the systematic approach employed, adherence to PRISMA-ScR guidelines, and prospective protocol registration strengthen the transparency and reproducibility of our findings.

## CONCLUSION

Cerebral myiasis, although rare, presents significant clinical challenges and can result in fatal outcomes, as demonstrated in this case. Given its rarity, often underestimated and occasionally mismanaged, there remains no clear consensus on optimal management. However, early diagnosis, prompt surgical debridement, targeted antibiotic therapy, and daily wound care are essential. Maintaining strict wound and patient hygiene is also a critical parameter for favorable clinical evolution. The treatment strategy employed highlights the need for a multidisciplinary approach and further research to establish standardized management protocols and improve patient outcomes.

## Data Availability

The complete anonymized dataset supporting the findings of this study is included within the article itself.
